# Implementing social accountability for contraceptive services: lessons from Uganda

**DOI:** 10.1186/s12905-020-01072-9

**Published:** 2020-10-12

**Authors:** Victoria Boydell, Nanono Nulu, Karen Hardee, Jill Gay

**Affiliations:** 1 Global Health Centre, Geneva Graduate Institute, Chemin Rigot 2, 1202 Geneva, Switzerland; 2 Department of Population Studies, Makerere University, Kampala, Uganda; 3 What Works Association, Washington, DC USA; 4 MIA, What Works Association, Washington, DC USA

**Keywords:** Gender, Social accountability, Contraception, Case studies, Uganda

## Abstract

**Background:**

Growing evidence shows that social accountability contributes to improving health care services, with much promise for addressing women’s barriers in contraceptive care. Yet little is known about how social accountability works in the often-complex context of sexual and reproductive health, particularly as sex and reproduction can be sensitive topics in the open and public formats typical of social accountability. This paper explores how social accountability operates in the highly gendered and complex context of contraceptive care.

**Methods:**

This exploratory research uses a case study approach to provide a more grounded understanding of how social accountability processes operate in the context of contraceptive information and services. We observed two social accountability projects that predominantly focused on contraceptive care in Uganda over a year. Five instruments were used to capture information from different source materials and multiple respondents. In total, one hundred and twenty-eight interviews were conducted and over 1000 pages of project documents were collected. Data were analyzed and compiled into four case studies that provide a thick description of how these two projects operated.

**Results:**

The case studies show the critical role of information, dialogue and negotiation in social accountability in the context of contraceptive care. Improved community and health system relationships, community empowerment, provider and health system responsiveness and enhanced availability and access to services were reported in both projects. There were also changes in how different actors related to themselves and to each other, and contraceptive care, a previously taboo topic, became a legitimate area for public dialogue.

**Conclusion:**

The study found that while social accountability in the context of contraceptive services is indeed sensitive, it can be a powerful tool to dissolving resistance to family planning and facilitating a more productive discourse on the topic.

## Background

Greater accountability is increasingly considered as central to improving health service delivery, patient experience and health outcomes in low- and middle-income countries [28, 59, 63]. In particular, social accountability, here defined as ongoing collective efforts to engage public institutions for accountability in the provision of public goods [26], has reportedly improved service provision and utilization, and health care provider behavior and responsiveness [5, 6, 22, 25, 40, 42]. Studies have also documented enhanced rights literacy, improved social cohesion, and trust and mutual collaboration between communities and health workers [22, 43–46]. Learnings from across the health sector suggest that social accountability addresses local barriers to health care, particularly those related to inequitable access and quality of care [28].

Service level barriers to quality contraceptive services, such as inconvenient hours and long waiting times, inaccurate and incomprehensible information, disrespectful and discriminatory treatment by service providers, along with untrained health care providers, lack of supplies and informal fees, negatively affect women’s confidence in and use of contraceptive services, thereby limiting their ability to exercise their reproductive rights [11, 17, 24, 47, 50, 62]. These conditions exist in some parts of Uganda, where women’s and men’s ability to freely decide on the number and spacing of their children – and have the information and means to do so – remains constrained after years of high-level political opposition [3, 27, 34–39, 49, 52, 53, 56].

Uganda’s modern contraceptive prevalence rate increased from 14% in 2000 to 35% in 2016 [54]. Yet, many married women (42%) still have an unmet need for contraceptives, particularly those living in rural settings, with lower incomes and less education [54]. Many women face a 40-min walk to public health facilities (3 to 5 km) and, upon arrival, their desired contraceptive services maybe unavailable due to stock outs or staff are not trained in providing different methods [27, 35, 39, 55]. Congestion in public facilities translate into overstretched staff and long waiting times that exacerbate opportunity costs and social risks [35, 56].

Women seeking contraceptive care in Uganda, particularly younger women, report poor treatment by health care providers and norms about age, marital status, parity and education influence how providers counsel clients [27, 36–38]. Though changing, social and gender norms continue to influence fertility preferences and shape social opposition to women’s access to and use of contraception [3, 2, 27, 34, 35, 37–39, 49, 52, 53]. Partner opposition, with the potential threat of verbal and/or physical abuse or abandonment, continue to underlie contraceptive non-use [35, 37].

Several studies have shown that social accountability can improve access to and use of contraceptive services [5, 22, 60]. For example, Gullo et al. [22] found an estimated increase of 57% in family planning use in Malawi following a community scorecard process, and Björkman and Svensson [5] found an increase of 22% in family planning use in Uganda after a report card process. These effects are surprising given that contraceptive care is often a political and personal topic that can be sensitive in the open discussions typical of social accountability. Little attention has been paid to understanding the mechanisms behind this relationship. This paper addresses the lack of understanding of how social accountability processes operate in highly gendered and complex context of contraceptive care [9, 10]. It presents exploratory research that uses descriptive case studies to provide a more grounded understanding of how social accountability processes works to improve access to family planning. We observed two social accountability projects focused on improving contraceptive care in Uganda.

## Methods

To address this research gap, we conducted exploratory research using a descriptive case study design in four districts of Uganda - two in Western Region and two in Central Region – over a one-year period. We used qualitative research methods to facilitate the collection of respondents’ experiences, opinions, and thoughts that can help better understand the relationship between social accountability and contraceptive care. A case study design allowed for rich and detailed accounts of the social accountability processes over time in each site, outlining the different perspectives and events and incorporating the relevant contextual and implementation information [51, 64].

The districts were selected based on the presence of ongoing social accountability projects focused on improving access to quality contraceptive services implemented by Reproductive Health Uganda (see Table 1). Reproductive Health Uganda (RHU) is a Ugandan non-governmental organization (NGO) with decades of experience in sexual and reproductive health service provision and advocacy throughout the country. Two projects were selected in Western and Central region and both aimed to strengthen the capacity of service users and civil society to hold those duty-bearers with the responsibility to provide contraceptive care to account for promised services.

**Table 1:** Demographic information for the study districts [53, 57]

Study district	Western Region		Eastern Region	
Study site	B1	B2	A1	A2
Total population	328,964	456,958	528,231	281,705
Percentage of women aged 15–19 who have begun childbearing	23.7%		8.8%	
Percent distribution of currently married women and sexually active unmarried women aged 15–49 using any modern method	42.1%		43.2%	
Unmet need for family planning	24.1%		19.9%	
Percentage of women aged 15–49 who had a live birth in the last 5 years and receive antenatal care from a skilled provider	98.1%		99.8%	
Percentage of women aged 15–49 who had a live birth delivered by a skilled provider in the last 5 years	77.3		70.7%	
Percentage of women and men aged 15–49 who have ever experienced sexual violence	23.1		22.7%	
Maternal mortality rate (per 1000 women)	0.74		0.801	

Both projects operationalized social accountability in different ways; one of the projects used what they called a ‘community dialogue’ approach and the other used a ‘community scorecard’ approach. The ‘community dialogue’ brought together groups of village members to identify the issues they faced when accessing family planning. Drawing on these insights, specially trained representatives (i.e. champions) from these groups interacted with local health authorities to advocate for change and reported back to the village groups. This cycle was repeated on a quarterly basis. In addition, the village participants engaged in health promotion for family planning work and set up self-help groups. The community dialogues were distinct from the community scorecard approach which directly brought together service users and service providers (both at the facility and in local health authorities) to jointly assess the issues underlying service delivery problems and find a common way of addressing them. The scorecard activities were implemented alongside activities to strengthen wider health sector accountability, such as improving human resources for health and district-wide strengthening of health facility committees. The activities focused on holding a range of health systems actors accountable, from the providers at the health facilities through to local health authorities. Table 2 provides a detailed summary of the projects’ objectives, program theories, key actors and approaches.

**Table 2:** Summary of the two social accountability projects

	Case A: (2014–2017) (A1 and A2)	Case B: (2014–2017) (B1 and B2)
**Sites**	26 sub-counties, two districts in Western Region	Six sub-counties, two districts, Central Region
**Description of Approach**	Community Scorecard	Community Dialogue
**Expected outcomes**	**Goal:** Improved quality, accessibility and availability of health and social services **Result 1:** Citizens demand improved quality of services **Result 2:** Civil Society Organizations (CSOs) effectively advocate for issues of citizens’ concern in health and social sectors **Result 3:** Institutional capacity of CSOs strengthened	**Goal:** Contribute to the strengthening of women’s reproductive health and rights in Uganda **Outcome:** By 2017, the demand for quality family planning services has increased by at least 4% in two districts **Outcome:** By 2017, Reproductive Health Uganda has increased participation in and influence on strategically selected local and national policy processes for the promotion of family planning
**Theory of change**	If citizens are empowered to act on their choices and take lead in advocating for change, THEN, they would believe and have confidence that they can hold their leaders accountable and influence them to change policies in their favor. This would motivate citizens to demand better services from their duty bearers. The persistent collective voice and actions from citizens and community structures would compel duty-bearers to respond by changing the necessary policies and taking other actions that lead to improvements in the accessibility, availability and quality of health and social services.	Empowered beneficiaries will take responsibility in advocating for sustainable change. Sustainable change will be attained through the rights holders knowing their rights and how to address these to achieve influence; creation of demand for FP in the community; advocacy involving women to hold duty bearers accountable; and, in time, improved access to reproductive health services.
**Key project actors at sub-district and village levels**	• CSOs • Fora for citizen engagement	• Village-level women only Pressure Groups (PG), Female Champions, Male Role Models (MRM) and women’s groups
**Social accountability approach**	Combined social audits and citizen report cards. Compiled service information from both service users and providers who are then supported to jointly identify priority issues and develop action plans to address them. The community scorecard activities were implemented alongside activities to strengthen wider health sector accountability, such as improving human resources for health and district-wide strengthening of health facility committees.	Interactive participatory communication. Participatory process of sharing information between people to help reach a mutual understanding and a workable solution.

Both projects worked within the decentralized public health service system in Uganda that is structured into national and regional hospitals, general hospitals, and into four levels within districts based on catchment areas [31]. Each higher level of the health systems provides more specialized functions and supervises of the lower level health facilities. Uganda’s (2006) *National Policy Guidelines and Service Standard* sets out the contraceptive services that should be provided by each level of provider down to the contraceptive pills provided by the community health workers [30].

### Study instruments

Five instruments were used to extract information from different source materials, to capture multiple points of view in all four sites (two per district) and provide a grounded description of project implementation. Table 3 describes each instrument used to collect data, the participants and the sample sizes for each component of data collection.

**Table 3:** Data collection instruments and sample sizes

Research instrument	Purpose	Sample			Sampling
		Total	Female	Male	
Document review	To understand program theories of change, intended outcomes and activity timelines, as well as reported implementation of activities and outcomes. Project staff prepared the reports representing narratives of the project from their perspective.	Not applicable (NA)	NA	NA	Over 1000 pages of documents were reviewed, including project activity reports, project planning documents, such as log frames/ results frameworks, baseline reports, annual reports to funders, and any evaluations undertaken.
Context mapping	To understand the prior experience of the community during the previous 3 years, as well as ongoing interventions related to social accountability and family planning. The context mapping was conducted during the first 2 months of the project.	21	10	11	Participants were purposively sampled. They were approached through a telephone call and interviewed in person at their workplace. They included district health officials and local non-governmental organization (NGO) staff.
In-depth interviews	To explore experiences and perceptions of activities in the social accountability process with community members, project staff and intervention participants, particularly in regard to family planning, over the year of observation.	73	36	37	Participants were conveniently sampled at one of 12 activities and included: district officials, sub-county and local leadership, health providers and officials, project champions; Community Based Organization (CBO) members, farmers, teachers, business people, male role models, church leaders, project staff. Participants were approached face-to-face to be interviewed after the activities in private location nearby.
Non-implementation interviews	As social accountability is a process over time, it was important to consider stoppages and delays. The interviews probed reasons and perceived impacts of such interruptions to further understand the realities of implementing social accountability.	9	3	6	Participants were purposively sampled based on project staff recommendations. They included project staff, district and local executive staff, health workers and project champions. There were approached through a telephone call and interviewed privately in their workplace.
Remedy and redress interviews	In-depth interviews and observations indicate instances where a change was reported and attributed to the projects. To examine these changes and unpack the mechanisms of change, interviews were conducted to understand how people thought a particular change came about.	25	16	9	Participants were purposively sampled through snowball technique based on their role in the reported change as suggested by the project staff. They included project champion, community mobilizer, male role model, project staff, health committee members, local executive official, health official, and CBO member. They were approached through a telephone call and interviewed in their workplace or private location. They were asked to describe their perceptions of why the change took place and what they believed to be the impacts.

### Data collection

Over 1000 pages of project documents were obtained from the project staff and include project plans, log frames/result frameworks, reporting and evaluation reports. The documents detailed project design and planning and were analyzed to examine what each project intended to achieve and how it intended to go about it.

Data were collected through in-depth interviews and observations over a year, starting when both projects had been implemented for at least a year. The in-depth interviews captured information on people’s experiences of the events, the core components, and the barriers and facilitators. The observations captured interactions, attitudes and behaviors of project participants’ during project activities and events. Eight data collectors (three men and five women, including two of the authors) conducted the interviews and the observations. Data collectors were post-graduate students with at least a university education, previous experience in conducting interviews in sexual and reproductive health and spoke the local language. Data collectors were recruited and hosted at RHU and received 3 days of training prior to data collection and a one-day refresher training halfway through the study. Interviews were conducted in the local language and on the whole, there were no existing relationships between the interviewers and respondents, with the exception of project staff who had arranged data collectors’ access to project activities. All respondents were interviewed in private at the project events or at their workplace; they were informed about the study goals in their local language and asked to provide written consent. Interview guides were developed with semi-structured questions and prompts (see Additional file 1). The interviews lasted about 1 h and were audio-recorded with the consent of the respondents. For the observations, field notes were completed by the researchers after attending the events. 

### Data analysis

One hundred and ninety three interviews were conducted with a range of respondents (see Table 3 for details on interview type, gender and sampling). Interviews were transcribed into English and each transcript was checked by a member of the research team. The transcripts were coded in Atlas.ti by four coders. Initial deductive codes were developed based on the definition of social accountability and the research question, and emergent codes were found through jointly coding four transcripts by respondent type. To ensure consistent application of the coding frame, the four coders jointly coded a further four transcripts, and the remaining transcripts were divided between them. Weekly calls were held between coders to review codes and the lead author checked the consistency between coders. Codes were organized under themes and by respondent type, and written into thematic reports, which were triangulated for each site.

Project documents and fieldnotes were analyzed together. Project documents were reviewed to develop a detailed chronology of how the project was implemented. The intended activities were compared against the reported ones, and against the data collectors’ observation fieldnotes. The authors (VB and JG) reviewed and extracted information about the projects from the collected documents.

To construct the case studies, the two sets of analysis were compiled into a case study format for each site, with two case studies for each social accountability project. Contrasting the case studies within and between the two projects highlighted the similarities and differences in how social accountability operated in each site, and helped to identify common findings across all the case studies. Case studies and findings for Western Region are designated A1 and A2, and those for Central Region are designated B1 and B2. Within 6 months of completion of data collection, workshops were held with respondents in each site to discuss and validate the research findings.

## Results

Case studies of each project are presented in narratives of how social accountability operated in the context of contraceptive care.

### Case study A: the community scorecard approach

Prior to the implementation of the project in both districts, there was limited awareness about family planning. There was open hostility to family planning with rumors abound about its detrimental side-effects such as cancer, fatigue, and weight gain. Given the project’s focus on wider health system strengthening, RHU only began to focus their activities on family planning in the second year of the project. RHU introduced the topic of family planning to the project’s existing health authority partners, champions and community organizations (CO) who had already been trained in community mobilization, monitoring and advocacy. RHU introduced the new focus on family planning by training their community based organization (CBO) partners on contraception and supporting them to identify gaps in contraceptive care locally and to integrate these issues into their existing work plans.

After the training, the CBO partners undertook extensive community mobilization on family planning alongside sensitizations about health rights. The health rights training was important because community members learnt about their entitlements and what standards of care they should expect. A local teacher said, *“You cannot solve the problem you don’t know”* (A2). Community members shared their new knowledge of entitlements with other community members. The project staff also worked with local religious leaders from different denominations to actively make positive statements about family planning to their congregations.

Community members recalled the family planning activities and stressed that family planning was something they had to ‘learn’, as a local champion explained:

*Family planning did not emerge from a community dialogue because we learnt about family planning …. even on Sundays [when I would] go to church and tell the Reverend that today I would like to teach family planning or maternal health. Now the Reverend also accepts it and gives me a few hours and I teach men, women and all the children when they have all come to church* (A1).

In tandem with these community focused activities, the project staff sensitized health providers and local health officials about health rights, accountability and family planning. Local health officials found this training helpful in their work. A local official explained, *“Actually it was useful because what they were telling us was more or less teaching us how to uplift our area, especially the communities, because there are very many questions or problems or challenges in our communities” (A2).*

After separate sensitization with both the community and the health systems actors, both groups came together in interface meeting to jointly identify issues, develop priorities and strategic actions to address them. Together they identified shared concerns about misconceptions surrounding family planning, commodity stock outs and untrained service providers and how to address them. The desired changes were then regularly monitored by the community.

The interactions generated mutual understanding, and empathy between community members and health system actors. Community members felt empowered that they could raise their voice, as a local church leader commented, *“It was important because what community people said, they got time to say things they don’t have [the ability to say] anywhere else” (A1).* A health care provider explained how these exchanges made her appreciate the communities’ voice:

*If there is no voice from the community, it may take forever to have a better policy or, if a policy is in place and the community doesn’t understand what that policy says or what they will benefit when that policy is implemented, then it may also be another hindrance in policy implementation. So that is where we say we are heading to, we may not be there, but we are somewhere, we have achieved a few milestones along the way (A1).*

Health system actors began to view community inputs as valuable and forwarded the joint concerns to sub-country and district authorities for further action. There was an emergent sense of collaboration between the community and the health system. A community member who was involved in one such initiative explained, *“I have been able to learn many things like working together as a group for the better of our community”* (A1).

Some facility-related issues, such as poor road access, limited water and electricity supplies, poor provider behavior and lack of security, were attended to locally without relying on assistance from local officials or NGOs. A community member explained this established practice:

*We as community members…decided to come together, make an effort to solve some of these problems on our own without having to wait for outside help, which should come in later, at least we need to do half of these things ourselves (A1).*

The project did not report on changes in contraceptive care because this was not part of its performance monitoring requirements. However, there were several changes attributed to the project that indirectly benefitted contraceptive care, such as the recruitment of new health care providers and changes in provider behavior (e.g. wearing of uniforms and posting of duty rosters). The project participants themselves reported increased awareness of health issues, including family planning; having more confidence in the health system, and felt that their local health care providers and local leaders listened and acted on the issues they raised. Though the project tended to focus on broader health systems strengthening rather than family planning, it was apparent that, over time, access to family planning became a legitimate concern for discussion in community forums.

### Case study B: community dialogue approach

There were frequent reports of social resistance and opposition to contraception in both districts where the project was implemented. A project champion explained, *“Women used to fear to come out in the open to demand a family planning method of their choice. They feared that if people knew they were using family planning, they would think they are a prostitute, that, along with their husband, is there another one (126:54)?”* There were misconceptions about how contraception worked and what kind of effects it had like causing foetal abnormalities, cancer and fibroids. Another champion explained how contraceptive use was influenced by gender norms: *“When they [women] get married, their bodies, including the sex part, traditionally belongs to the man. The man decides when to have sex, when to have children and how many children”* (B1).

In contrast with case study A, family planning was integral from the outset of the project. There was ongoing sensitization of both communities and local health systems actors on family planning to dispel myths and tackle social resistance. The sensitization was conducted through women’s pressure groups, male role model groups, radio programs, couple counseling seminars and workshops among sub-county leaders. These activities were key, as a champion said, to *“Clearing the image that people were painting of family planning” (B1).*

RHU trained community champions (local women with social standing who had experience and skills in negotiating with leaders) and supported the formation of women-only “pressure groups (PG)” in the project villages. The champions and PG members met every 2 months to learn about family planning, prioritize which access barriers to address and report on the number of people they sensitized about family planning. Often RHU sent in their own service providers to conduct the training as a high degree of technical knowledge was required. The barriers they discussed ranged from transport costs, male resistance, myths and misconceptions, religious opposition, disrespectful providers and lack of information in local languages.

The newly formed groups organized the meetings, developed their activities and led sensitization about family planning in person or at social gatherings. To attract members, RHU launched income-generating activities in catering, animal rearing and savings. The activities proved so successful that these groups registered with the local authorities as independent organization so they could access additional funds.

As male opposition was considered a barrier to accessing family planning services, the project supported couple counseling and the formation of male role models (MRM). Male role models were local men with social standing trained to promote family planning with other men, both individually and in groups.

Participants in these community meetings thought they were catalytic and helped them develop personally and gain self-worth. One MRM said, *“Every time you are with a person, you bring a good idea, and someone claps for it, it means it is of value”* (B1). A youth leader felt that they could make a difference, *“What did I learn? I learned that even though you are small, you can make an impact”* (B1). A project champion observed these changes:

*The people who attended were so free, why am saying that, when these things started there were people who had low self-esteem most especially pressure groups they used to fear, when they would see champions they would fear. But slowly we started visiting them in their activities, we would be called, and we go to explain to them where they do not understand. So you look and see a champion sitting here, a pressure group sitting there, but it was never there (B1).*

Members appreciated learning from each other and working together, as one community member said:

*Everyone was getting a chance. You know in that group we have health workers, we have village women, teachers … so to say there is also a class of people that do government work and also a class of women who do their own work in the villages but we were in the meeting and everyone was being given a chance to talk* (B2).

Once the barriers to contraceptive care were identified in the community meetings, the project champions and RHU met with local authorities to advocate for change. For example, a barrier identified was that women could not seek contraceptive services because of the prohibitively high transport costs or fees charged during private outreach programs. The champions and RHU, therefore, advocated with the district health authorities to integrate family planning into ongoing immunization outreach. These advocacy efforts were relatively successful, with sub-country and district funds committed to family planning in the first year of the project. In the subsequent years, the advocacy focused on ensuring the committed funds were released and used as intended.

In the meetings with local authorities, people with different backgrounds shared their experiences and ideas, and learned from and about each other. Local officials came to value inputs from the surrounding community, *“We lack such information and yet people run to us to provide a solution to them, so we need such information more than ever*” (B2). Officials also appreciated learning about family planning and shared this with their constituencies, *“My role was to participate, to see what family planning is, to understand what they have taught so that I can go back and spread the gospel to my people who have not got this chance” (B1).*

Over time, however, participants reported divisions among the community actors. The champions were treated differently; they received additional training, and directly engaged with officials that the other community groups did not. This difference was palpable, as a pressure group member stated: *“We have the champions, those that are higher than us, because for us, we are lower as PG members”* (B1). These social differences among community members played out in what was included in the dialogues with health system actors; quality of care issues identified by the Pressure Group received less attention than policy related barriers, particularly those with budgetary implications. Over the course of the study, the MRMs began mobilizing for other projects they were involved in, and the PGs set up autonomous groups with funding from other sources.

The project successfully secured several budget lines for family planning, many self-sustaining women’s groups were formed, and the district-wide platform to coordinate family planning was established. There was a more positive attitude towards family planning, and it had become a legitimate public concern. One champion said,

*There has been some change, because back then we had our people who never wanted to hear anything about family planning because they were taking it in a very different way, they would say stop-stop don’t even tell us, so that is what has been on ground. But now they even visit us and also us we visit them after, so we see a great change because people are now involved in family planning, something that had never happened in the past (B2).*

## Discussion

With the increased attention to social accountability and making health systems more accountable, two projects aimed at improving access to quality contraceptive services implemented by RHU in Uganda offer important lessons on how social accountability operates in relation contraceptive services. Both projects, like other studies of social accountability, reported improvements in community and health system relationships, community empowerment, and provider and health system responsiveness to community concerns [22, 43]. These changes were generated by actors viewing themselves and each other differently, whether by considering themselves as agents of change or taking the views from others as valuable. In addition, over the course of both projects, family planning went from being taboo to an appropriate topic for public dialogue. Much like other accounts of social accountability in the context of reproductive health and maternal health, across the four case studies, information, dialogue and negotiation were central to the change process [20, 23]. The case studies provided grounded examples of information, dialogue and negotiation that can inform policy and practice.

### Information

Social accountability in the context of contraceptive care is more than improving access and quality; it entails bringing stigmatized or unacknowledged concerns to the surface and making silenced issues legitimate areas of public concern. In both project sites, contraception was considered a ‘woman’s issue’ and/or source of fear and social anxiety. This required a special programmatic emphasis on changing the associations surrounding contraception. RHU, an external actor that introduced the topic, was described as bringing in ‘new knowledge’ into the community. Wide-ranging efforts (such as small scale sensitization, working with religious leaders to make positive statements, and enrolling male role models to work throughout the villages) were used to challenge popular perceptions regarding contraception, including emphasizing its social and health benefits, and dislodging misconceptions and social opposition held by community members, health care providers, and duty-bearers. Efforts geared towards making an issue a legitimate area of public concern are a distinctive feature of social accountability in the context of contraceptive care.

Raising awareness and sharing information itself was insufficient to prompt change in either project sites; as others have argued, it is critical that health systems actors are willing and able to respond to community demands [16, 18, 26]. Viewing people’s claims as legitimate and showing “receptivity to the ideas and concerns raised by citizens by implementing changes to the decision-making or management structure, culture, policies or practices ([32]: 130)” are central to bringing about change [29, 32]. In the case studies highlighted in this study, health system actors were actively supported to overcome their own misconceptions both about contraception and about community engagement. Only then were the key stakeholders in contraceptive care open to listen to community needs and preferences [21, 23, 41]. Yet, the capacity and willingness of duty-bearers often go unrecognized [20, 29, 41, 43]. The need to support health care actors in social accountability was not reflected in the projects’ documents which treated responsiveness as an outcome rather than as part of the process. In both sites, however, project participants worked with local officials and health sector actors to raise their awareness about contraception, to actively value the concerns coming from the community as well as to improve their knowledge about the health system and their own roles in it.

### Dialogue

In the social accountability canon, dialogues are a central mechanism to counteract the hierarchical nature of the health services [14, 20, 22, 23, 41, 43–45]. Dialogues transform both how people perceive themselves and their ability to effect change, as well as how they perceive and interact with others, particularly those in socially advantaged and/or privileged positions. The community-level groups, including those initiated by the project and those that grew out of the projects, provided spaces for both women and men to reflect on issues that affected their reproductive health and to recognize them as shared concerns; this created a sense of agency and solidarity that translated into the dialogues and negotiations with health care providers and health officials. In these groups, people felt they could speak, that they were heard and valued, and that personal capabilities were expanded from working together. They increasingly viewed themselves as active and able to bring about change themselves; change was not something done for them.

The projects supported dialogues between people from different backgrounds and positions (from community members, community representatives, project staff, health care providers and local health officials) and offered new ways of interacting. Scott et al. [44] suggested that these kinds of dialogues are novel spaces where people who do not ordinarily come together meet and the normal rules of interaction are momentarily suspended. In these ‘safe spaces’, people are encouraged to reflect on their biases and assumptions, realize their own limitations and express their own constraints and frustrations [41, 44]. As other studies of social accountability found, these are opportunities for bi-directional information sharing, for expressing grievances and priorities, for official explanations of policies and responding to concerns, and for co-producing priorities and ways to address them [6, 21, 22, 41, 43].

In a context where there is social resistance to contraception, with reports of social and physical opposition from partners, parents, health care providers, religious leaders, and the limited knowledge and information about contraceptives, the dialogues do something more than challenging hierarchies. In both case studies highlighted in this paper, the dialogues played a critical role in sharing more positive ideas and information about contraception. The importance of small-scale social networks in increasing the local acceptability of family planning is well documented [4, 8, 33]. Demographers have noted that through social networks, local influencers facilitate discussions about ‘inappropriate topics’, increase exposure to positive experiences and positive outcome expectations of family planning, and facilitate opportunities to assess the relative benefits, all of which contribute to a more supportive environment [19, 58]. For women who may experience social isolation or internalize harmful social norms, forming local groups provides critical social support, confidence-building and self-efficacy in seeking family planning services [12, 13, 15]. Dialogues play several critical functions regarding how social accountability operates in contraceptive care such as supporting local solidarities to challenge hierarchies, and expanding local acceptability of family planning, particularly where there is social opposition.

### Negotiation

Dialogues can lead to new alliances in which communities and health system actors work together to negotiate with higher level authorities to bring about change. In both projects, by the end of the study, community inputs were increasingly valued by health system actors and were actively sought to be included in districts and at sub-county planning and budget meetings, district management meetings and health sector committee meetings. In addition, local health actors worked with community partners to develop strategies to ensure the implementation of policies locally, as well as upstream to press senior officials to act.

In the two projects studied, the role of community representatives (the champions in the community dialogue approach or sub-contracted local community groups in the community scorecard model) was notable. People with local social stature and strong networks were strategically leveraged to enhance the credibility of efforts and to help legitimize the demands of those in less socially advantageous positions. Scholars working in the field of social accountability have identified the legitimacy of groups and their demands as a critical factor in confronting unequal relationships behind inequitable access to care [14, 20, 41]. Marginalized groups often draw on the social capital generated through connections with local community groups, non-governmental organizations and local personalities to help legitimise their demands [41]. Yet findings from this study show that the participatory mechanisms were managed by more privileged participants who promoted what they thought was most important, while those more vulnerable were side-stepped.

In both cases, the social standing of the community representatives played a role in legitimizing women’s demands, and acted as a safe outlet for women who felt social constraints in what they could say and do for fear of social censure. Given the resistance to family planning in the study setting, navigating these gendered norms and hierarchies may be an unavoidable necessity and essential for women in the community. Intimate partner violence as a result of contraceptive use is well-documented globally and in Uganda and many  women resort to covert use of contraception [1, 7, 48, 61]. Given the social and physical risks associated with contraceptive use, working with intermediaries may be a safer way to raise and discuss sensitive issues and deflect the risks posed to individual women to demand for quality contraceptive information and services. The findings from this study show that the social complexities of communities, at a minimum, were recognised in the programme design.

The findings of this study show that contraceptive care has some unique features. First, early in the process, special programmatic efforts were required to make contraceptive a legitimate topic of community concern among community and health system actors. This study shows that sexual and reproductive health decisions,  including access to family planning, are regulated by local norms and expectations around gender, marriage and kinship, and are often considered as the private domain of the family. Its private connotations may prevent it being raised in public forums and requires special endeavors to make it an acceptable topic for public discussions. This study found that positive public conversations about contraception set the grounds for more open conversations about barriers and solutions to information and services.

The findings further suggest that another unique dimension is the need to strike a balance between personal safety and participation in dialogues and negotiation. The social and physical risks around publicly discussing sexual and reproductive decisions are unmistakable and require careful attention. The strategic use of community representatives who were socially advantaged and potentially less exposed to the social and physical risks presented an innovative solution in both project sites. Yet, in both cases, the alliance between the community groups and their community representatives was short lived, as they split towards the end of the project to pursue their own interests. Social accountability approaches are likely to be sustainable, particularly those that involve changing local perceptions and acceptability of contraception, and supporting the formation of trained local solidarity groups groups, which could extend beyond the lifespan of the project.

### Limitations

This study has certain limitations. First, the study districts were purposively selected because they were conducting social accountability projects to improve accesss to family planning and both were implemented by RHU. RHU has been working on sexual and reproductive health and rights in Uganda for decades and is therefore not representative of all NGOs doing similar work. Second, there was a potential bias towards positive responses. The interviewers were recruited by RHU and could have been perceived as RHU staff, and respondents were recruited during the activities or based on recommendations from project staff. This potential bias was addressed by including respondents beyond project implementers and participants. Furthermore, bias was minimized through triangulating the sources when interpreting data and through validation of the findings with the respondents at the end of the study. Where the responses differed, the differences were described in the analysis and interpreted by the coders in the context of other findings. Third, the paper reports the changes as perceived by the respondents based on one year of project implementation, rather than an impact evaluation. The study design had originally included measuring contraceptive uptake, but there was no suitable program data or service delivery data available to assess this outcome. In crafting a concise narrative it was necessary to leave out some data. One such dimension was the deep frustration about the delays in funding that resulted in stoppages in implementation of activities which affected the trust, morale, credibility, and opportunities that the projects had been building upon.

## Conclusion

The case studies included in this paper provide important insights into how social accountability works in the often-sensitive context of sexual and reproductive health care that are cogent for other settings and for socially and politically complex issues. We found that while social accountability in the context of contraceptive services is indeed sensitive, it can be a powerful tool to dissolving resistance to family planning and facilitating a more productive discourse on the topic. Findings from the case studies show that social accountability can generate common causes and lead to slow-burn transformative changes, through information, dialogue and negotiation, that can improve health services and wider social dynamics.

## Supplementary information

**Additional file 1.** Interview Guides.

## Data Availability

The dataset analyzed during the current study will be available at the USAID Development Data Library in January 2021, https://www.usaid.gov/data. They will also be available from the corresponding author on reasonable request.

## Abbreviations

ABH: Advocacy for Better Health
CBO: Community Based Organizations
CO: Community Organizers
CSO: Civil Society Organization
ICF: ICF International Ltd.
MoH: Ministry of Health
MRM: Male Role Model
NA: Not applicable
NGO: Non-Governmental Organization
PG: Pressure Group
RHU: Reproductive Health Uganda
UBOS: Uganda Bureau of Statistics
UHRC: Uganda Human Rights Commission
WRAP: Women Regional Advocacy Project
