# Supplementary material for: Implementing social accountability for contraceptive services: lessons from Uganda
Source: BMC Womens Health. 2020 Oct 12;20:228. doi: 10.1186/s12905-020-01072-9 (PMC7549211; doi:10.1186/s12905-020-01072-9)
Supplement: Supplementary file 1 — Additional file 1. Interview Guides. [file 12905_2020_1072_MOESM1_ESM.docx]

## Interview Guide: Supplementary File 1

## Context Mapping Interview Guide

*I would like to begin our conversation by asking some questions about you*

- What is your job? How long have you been in this role?
- Can you tell me more general information about your background or training?
- Are you and your family from this area? If not, where are you from?

*I would like to have a better understanding of the community engagement and monitoring activities that have taken place here over the last three years*:

- Can you recall any activities that involved the community being engaged in, monitoring or influencing local decision making about the services and monitoring activities:
  - What was the purpose of the activity?
  - What did the activity involve?
  - Who implemented the project?
  - What were the results, if any, and why?
  - What were the challenges or barriers?
- How did the target decision makers respond?

*I would like to have a better understanding what activities have been taking place to improving family planning and reproductive health services here over the last three years:*

- Can you recall any activities *to improving family planning and reproductive health services, both public and private:*
  - What was the purpose of the activity?
  - What did the activity involve?
  - Who was implementing the activity?
  - What in your opinion were the results, if any, and why?
  - What were the challenges or barriers?

## In-Depth Interview Interview Guide

| **Participant Group** | **Tick Type:** | **Question Schedule to use** |
| --- | --- | --- |
| Participants - Citizens | - Participant –unmarried woman - Participant – unmarried man - Participant –married woman - Participant – married man | Question Schedule One |
| Participant - Official | - District Health Officer - District Secretary of Health - District Community Health Officer - Member of Health Unit Management Committee | Question Schedule Two |
| Participant – Service Providers | - Service provider - Village Health Team - Community based distributors | Question Schedule Three |
| Participant - Partners | - CSO partner - NGO Forum member - Implementation Partner | Question Schedule Four |
| Participant – Project staff | - District Project Coordinator - Project coordinator Kampala | Question Schedule Five |

Please tick the type of event being observed:

- Civic education or rights awareness of citizen
- Data collections tools and/or strategy development (political mapping, data collection, stakeholder analysis, power analysis, budget and policy making; community needs assessment, monitoring service delivery, etc.)
- Building or strengthening local partnership with CSOs, CBOS and Implementation partners
- Community mobilization and strategy/resolution meeting
- CSO led training of champions, media, community researchers
- Development of accountability tools (scorecard, community resolution, presentations)
- Engaging decision makers – one-on-one meetings; interface meetings, public hearings, created spaces (stakeholder forums) or invited spaces (District management; council meetings, budget meetings, Health Facility Management meetings
- Quarterly Project Meeting
- Other Events: (define)

**Schedule One – Citizens**

Section One: General Information

*I would like to begin our conversation by asking some questions about you*

- What is your job? How long have you been in this role?
- Are you and your family from this area? If not, where are you from?

Section Two: Description of the event

*I would like to ask you some questions about the event you just attended?*

The Event:

- Can you describe to me event you just attended? Your overall impressions?
- What was the stated purpose of the event? Was there an informal purpose for the event?
- What was the structure of the event, e.g. introduction of the objectives and participations, presentation of information, discussion of information and planning next steps)
- Was it interesting or relevant to you?

Your Role:

- What was your role in this event?
- Why did you get involved this? What motivated you to get involved?
- Where you involved in the preparation in any way or prepared before coming here today?

People and interactions

- Who was at the event? What did they do?
- Can you describe how people were involved?
- How they interacted with each other (few people participated, people were bored etc.)
- Was there chance for everyone to speak or did only a few people speak?
- Who do you think should have been at this event and was not?

Outcomes

- What was the outcome and why do you think it is important?
- What were next steps of the event? How were they decided?
- Do you think people will change what they normally do because of this event?
- Will you change what you normally because of what you heard here today?
- Can you think of another way that the same change could happen?
- How do you think this will change the family planning services that are provided here?

Empowerment

- What do you understand by the term empowerment of citizens in this project?
- Has this project empowered citizens? If yes, how?
- Could you please give examples of how this project has empowered the citizens?
- Do you believe citizens are mobilized to access and demand FP services? If yes, what methods are used to mobilize them?
- What things are likely to continue the way they are after ABH/WRAP Project has ended? Probe for what will happen on integrating family planning in community activities, District budgets, etc

**Schedule Two - Officials**

Section One: General Information

*I would like to begin our conversation by asking some questions about you*

- What is your job? How long have you been in this role?
- Are you and your family from this area? If not, where are you from?

Section Two: Description of the event

*I would like to ask you some questions about the event you just attended?*

The Event:

- Can you describe to me event you just attended? Your overall impressions?
- What was the stated purpose of the event? Was there an informal purpose for the event?
- What was the structure of the event, e.g. introduction of the objectives and participations, presentation of information, discussion of information and planning next steps)
- Was it interesting or relevant to you?

Your Role:

- What was your role in this event?
- Why did you get involved this? What motivated you to get involved?
- Where you involved in the preparation in any way or prepared before coming here today?

People and interactions

- Who was at the event? What did they do?
- Can you describe how people were involved?
- How they interacted with each other (few people participated, people were bored etc.)
- Was there chance for everyone to speak or did only a few people speak?
- Who do you think should have been at this event and was not?

Outcomes

- What was the outcome and why do you think it is important?
- What are next steps of the event? How were they decided?
- Do you think people will change what they normally do because of this event?
- Will you change what you normally because of what you heard here today?
- Can you think of other ways that the same change could happen?
- How do you think this will change the family planning services that are provided here?

Empowerment

- What do you understand by the term empowerment of citizens in this project?
- Could you please explain how this project has empowered the citizens?
- Could you please give examples of how this project has empowered citizens?
- Do you believe citizens are mobilized to access and demand FP services? If yes, what methods are used to mobilize them?
- What things are likely to continue the way they are after ABH/WRAP Project has ended? Probe for what will happen on integrating family planning in community activities, District budgets, etc

**Schedule Three – Services Providers**

Section One: General Information

*I would like to begin our conversation by asking some questions about you*

- What is your job? How long have you been in this role?
- Are you and your family from this area? If not, where are you from?

Section Two: Description of the event

*I would like to ask you some questions about the event you just attended?*

The Event:

- Can you describe to me event you just attended? Your overall impressions?
- What was the stated purpose of the event? Was there an informal purpose for the event?
- What was the structure of the event, e.g. introduction of the objectives and participations, presentation of information, discussion of information and planning next steps)
- Was it interesting or relevant to you?

Your Role:

- What was your role in this event?
- Why did you get involved this? What motivated you to get involved?
- Where you involved in the preparation in any way or prepared before coming here today?

People and interactions

- Who was at the event? What did they do?
- Can you describe how people were involved?
- How they interacted with each other (few people participated, people were bored etc.)
- Was there chance for everyone to speak or did only a few people speak?
- Who do you think should have been at this event and was not?

Outcomes

- What was the outcome and why do you think it is important?
- What were next steps of the event? How were they decided?
- Do you think people will change what they normally do because of this event?
- Will you change what you normally because of what you heard here today?
- Can you think of other ways that the same change could happen?
- How do you think this will change the family planning services that are provided here?

Empowerment:

- What do you understand by the term empowerment of citizens in this project?
- Has this project empowered citizens? If yes, how?
- Could you please give examples of how this project has empowered the citizens?
- Do you believe citizens are mobilized to access and demand FP services? If yes, what methods are used to mobilize them?
- What things are likely to continue the way they are after ABH/WRAP Project has ended? Probe for what will happen on integrating family planning in community activities, District budgets, etc

**Schedule Four - Project Partners**

Section One: General Information

*I would like to begin our conversation by asking some questions about you*

- What is your job? How long have you been in this role?
- Are you and your family from this area? If not, where are you from?

Section Two: Description of the event

*I would like to ask you some questions about the event you just attended?*

The Event:

- Can you describe to me event you just attended? Your overall impressions?
- What was the stated purpose of the event? Was there an informal purpose for the event?
- What was the structure of the event, e.g. introduction of the objectives and participations, presentation of information, discussion of information and planning next steps)
- Was it interesting or relevant to you?

Your Role:

- What was your role in this event?
- Why did you get involved this? What motivated you to get involved?
- Where you involved in the preparation in any way or prepared before coming here today?

People and interactions

- Who was at the event? What did they do?
- Can you describe how people were involved?
- How they interacted with each other (few people participated, people were bored etc.)
- Was there chance for everyone to speak or did only a few people speak?
- Who do you think should have been at this event and was not?

Outcomes

- What was the outcome and why do you think it is important?
- What were next steps of the event? How were they decided?
- Do you think people will change what they normally do because of this event?
- Will you change what you normally because of what you heard here today?
- Can you think of other ways that the same change could happen?
- How do you think this will change the family planning services that are provided here?

Empowerment:

- What do you understand by the term empowerment of citizens in this project?
- Has this project empowered citizens? If yes, how?
- Could you please give examples of how this project has empowered the citizens?
- Do you believe citizens are mobilized to access and demand FP services? If yes, what methods are used to mobilize them?
- What things are likely to continue the way they are after ABH/WRAP Project has ended? Probe for what will happen on integrating family planning in community activities, District budgets, etc

**Schedule Five - Project Implementers**

Section One: General Information

*I would like to begin our conversation by asking some questions about you*

- What is your job? How long have you been in this role?
- Are you and your family from this area? If not, where are you from?

Section Two: Description of the event

*I would like to ask you some questions about the event you just attended?*

The Event:

- Can you describe to me event you just attended? Your overall impressions?
- Was this event what was originally intended?
- What was the stated purpose of the event? Was there an informal purpose for the event?
- What was the structure of the event, e.g. introduction of the objectives and participations, presentation of information, discussion of information and planning next steps)
- Was it interesting or relevant to you?

Your Role:

- What was your role in this event?
- Why did you get involved this? What motivated you to get involved?
- Where you involved in the preparation in any way or prepared before coming here today?

People and interactions

- Who was at the event? What did they do?
- Can you describe how people were involved?
- How they interacted with each other (few people participated, people were bored etc.)
- Was there chance for everyone to speak or did only a few people speak?
- Who do you think should have been at this event and was not?

Outcomes

- What was the outcome and why do you think it is important?
- What were the next steps of the event? How were they decided?
- Do you think people will change what they normally do because of this event?
- Will you change what you normally because of what you heard here today?
- Can you think of other ways that the same change could happen?
- How do you think this will change the family planning services that are provided here?
- What do you understand by the term empowerment of citizens in this project?
- Has this project empowered citizens? If yes, how?
- Could you please give examples of how this project has empowered the citizens?
- Do you believe citizens are mobilized to access and demand FP services? If yes, what methods are used to mobilize them?
- What things are likely to continue the way they are after ABH/WRAP Project has ended? Probe for what will happen on integrating family planning in community activities, District budgets, etc

## Remedy and Redress Interview Schedule

*Section One: General Information:*

*I would like to better understand who you are and how you have been involved with the project intervention. I would like to begin by asking you some questions about yourself:*

- What is your role in the project?
- Why did you get involved?
- Are you from this area?

*Section Two: Description of the project thus far*

*I would like to better understand the activities related to the project so far and your views on what has taken place.*

- Can you describe what has been happening in the project?
- What do you enjoy the most?
- What do you think the other participants enjoy?
- How do you think the program achieves its goals?
- Have there been any challenges or problems with the project?

*Section Three: Moment of remedy and redress*

*I would like to better understand times when changes have taken place and are related to the intervention.*

- What have been the issues the community has raised?
- What have been the issues have you yourself been working on?
- What did you/they do?
- What were the actions?
- Did anything happen as a result?
- Have you seen any changes?
- If you have seen changes, why and how do you think they happened?
- What do you think the ‘magic ingredients’ are for changes like this?
- Have the duty bearers done anything specific as a result of the program?
- What do you the best way to achieve change is?

## Non-implementation interview schedule

**Knowledge of the pauses in the project:**

- How have you been involved in the project? Probe for implementation of activities or support to the project).
- Have there been any changes to the original program delivery plan for the project? (Probe: What were they?
- Can you tell me about the pauses in the project activities? (Probe: cause of pause and length of pause?)
- Have any alternative arrangements been made as a result of these unanticipated changes to project delivery?

**Activities / events/ meetings that were missed due to pauses:**

- Were there any activities or key project aspects that have not been implemented due to pauses or changes in the implementation plan?
- If yes, what were the objectives of this activity?
- Who were you expecting to participate in the activity that did not take place?
- What issues were going to be discussed at these meeting(s) or event(s)?

**Impact:**

- What do you believe are the implications of the delay of the activity for the participants? (e.g. lost of interest, lack of benefits, lack of momentum etc)
- Do you think these changes will impact the overall project result? How?
  - Probe: What types of information were not shared between community members, health providers and district officials due to pauses in the project?
- What key activities do you think were missed as a result of these changes/ pauses?

**Charting Implementation**

During this part of the interview the researcher will attempt to chart the original project timelines against what has actually taken place. Below is an empty timeline. Discuss with the research participant what the original time line was, or if possible, learn this prior to the interview and chart is on the upper part of the timeline tool.

List the activities in the boxes and mark when they took place on the timeline. Add any additional notes at the bottom of the page. It might be useful to number the activities.
